# Motivational Factors in the Typical Display of Humor and Creative Potential: The Case of Malevolent Creativity

**DOI:** 10.3389/fpsyg.2020.01213

**Published:** 2020-06-23

**Authors:** Corinna M. Perchtold-Stefan, Andreas Fink, Christian Rominger, Ilona Papousek

**Affiliations:** Department of Psychology, University of Graz, Graz, Austria

**Keywords:** humor, malevolent creativity, comic styles, latent goals, social goals

## Abstract

Research is still disputing if an individual’s use of humor in everyday life is also indicative of his or her creative potential. To date, the focus has been mainly restricted to shared cognitive factors, while motivational aspects that may link the production of humor and of creative ideas have been largely neglected. Humor motivation implicates latent social goals the creator pursues through the use of humor. These goals can be benign or more malicious and manifest in an individual’s typical display of comic styles. While often overlooked, creativity often serves social functions as well, especially in common everyday situations. Similar to humor, creativity is typically regarded as beneficial for individuals and society. Yet, creative ideas may also originate from less prosocial goals. This is reflected in the concept of malevolent creativity, where novel ideas are generated to deliberately harm others. The present study investigated individuals’ typical display of humor, differentiated in eight distinct comic styles in relation to their productivity in a behavioral test for malevolent creativity and general creative potential (*n* = 106). Individuals with higher scores on comic styles that are affiliated with malicious interpersonal goals – such as hurting or upsetting others or demonstrating superiority over others – were more fluent in producing malevolent creative ideas in the malevolent creativity test. This finding shows that individual differences in humor motivation relate to the capacity of coming up with relevant creative ideas also outside the domain of humor. The pattern of relationships between humor motivation and general creative potential differed from that of malicious creativity and implied the comic style “wit” only, primarily adding to the notion of shared cognitive processes in the production of humor and creative ideas. The study offers a novel perspective for how the inclusion of motivational factors that are inherent to conceptualizations of humor may also benefit creativity research.

## Introduction

Often regarded as pinnacles of human evolution, parallels between the use of humor and creativity have been drawn for decades. From earlier theoretical accounts and reports of positive associations between individuals’ broad “sense of humor” and their creative potential (e.g., Koestler, 1964; Galloway, 1994; O’Quin and Derks, 1997), modern scientific investigations have attended to more fine-grained links of creativity with specific aspects of humor production (Amir and Biederman, 2016; Kellner and Benedek, 2017; Papousek et al., 2019). So far, this research was strongly focused on overlaps of cognitive processes implicated in creative ideation and the creation of humor. Together, there is much evidence that humor production and creative ideation share fundamental cognitive properties. Both require breaking with conventional mental routines and linking remotely related concepts in unprecedented and surprising ways (e.g., Kozbelt and Nishioka, 2010; O’Quin and Derks, 2011; Benedek et al., 2012; Fink and Benedek, 2014; Luft et al., 2018; Chen et al., 2019; Ruch and Heintz, 2016).

However, beyond its apparent relation to creative cognition, humor creation implicates being creative in the social domain (Goodchilds and Smith, 1964). Humor is typically used in interpersonal situations, where it can satisfy certain social functions and goals (Long and Graesser, 1988; Martin, 2007; Kuipers, 2008). This social aspect of humor creation is related to humor motivation. The motivation to produce humor is regarded as the first stage in the process of humor creation, which is followed by the cognitive process of producing humor and, finally, humor communication (Feingold and Mazzella, 1993). Humor motivation is conceptually distinct from cognitive ability aspects of humor production and denotes why the humor is produced; that is, which social goals are pursued through the use of humor (Feingold and Mazzella, 1993; Ruch and Heintz, 2016). Humor can serve manifold social goals, some of which are benign, such as the formation, enhancement, and maintenance of social relationships, while others are malicious and include manipulative control, status enhancement, or ostracism of out-group members (e.g.,Weisfeld, 1993; Martin, 2007; Ferguson and Ford, 2008; Davies, 2009; Li et al., 2009; Proyer et al., 2012; Curry and Dunbar, 2013; Zeigler-Hill et al., 2013; Kashdan et al., 2014; Lackner et al., 2019). The social goals related to the use of humor are most probably “latent” goals, which can motivate action and direct behavior outside of people’s awareness and are thought to be implicated in many aspects of social life (Austin and Vancouver, 1996; Custers and Aarts, 2005; Bargh, 2006).

At the trait level, individuals’ latent interpersonal goals manifest in their typical comic styles (Papousek et al., 2017). Comic styles may provide insights into the ways in which humor is typically displayed in social interactions. Ruch et al. (2018) proposed a model of eight comic styles that cover specific ways in which people engage in humor in their everyday lives, which include but are not limited to, interindividual differences in the comprehension, appreciation, and production of humor. The model was transposed into the Comic Style Markers (CSM) questionnaire, which was extensively psychometrically tested and validated (Ruch et al., 2018; Heintz, 2019). The eight fine-grained comic styles cluster into broader groups: One cluster includes “dark,” mockery-related forms of humor, with sarcasm and cynicism at its core and implying latent malicious, mean-spirited goals and intentions of hurting or upsetting other people or demonstrating superiority. Other comic styles represent forms of humor clearly implicating benevolent interpersonal goals (“benevolent humor”) or convey mixed or neutral social goals such as nonsense humor or the use of clever and spontaneous wordplays (“wit”; see also Papousek et al., 2017; Heintz, 2019; Hofmann et al., 2019). In sum, latent malicious social goals seem to dominate in individuals with a greater inclination toward humor characterized by sarcasm, cynicism, and irony. Conversely, benign interpersonal goals seem to dominate in the comic style of benevolent humor. This was empirically confirmed in a study testing specific brain responses indicating the brain’s positive or rewarding appraisal of other people’s affect expressions that signaled that those people were hurt or cheered up (Papousek et al., 2017).

A recent study in which participants created humor in the form of humorous reinterpretations of threatening situations confirmed effects of humor motivation – mirrored in the comic styles – on actual humor production. Individuals with higher scores on dark comic styles (self-reported on the CSM) produced greater quantities of ideas implying malicious (disparaging) forms of humor in the behavioral performance test (Perchtold et al., 2019). Participants with greater typical use of more light or neutral comic styles, in particular wit, also produced a higher quantity and quality (funniness, originality) of humorous ideas in a nonsocial cartoon caption task (Heintz, 2019). These findings suggest that individual differences in humor motivation, to some extent, translate to the productivity in creating (certain types of) humor. Importantly, rather than a relative preference for one or the other comic style, the model and questionnaire capture the inclination to display particular types of humor separately. That is, the comic styles are not mutually exclusive. Some individuals may engage in several or even all forms of humor to a greater extent than others. Thus, there is also a higher-order (general) factor of humor motivation (Ruch et al., 2018), which typically manifests in moderately positive intercorrelations among the individual CSM scales (Ruch et al., 2018; Hofmann et al., 2019; Mendiburo-Seguel and Heintz, 2019).

While largely neglected so far, creativity often serves social functions as well, especially in every day, real-life situations. Hence, it was argued that similar social processes may be involved in humor production and non-humorous creative ideation (O’Quin and Derks, 1997). Traditionally, creativity is assumed to be a virtuous, highly desirable ability, fueling innovation and progress for the greater good (e.g., Stein, 1953; Sternberg, 2010; also see Fink and Benedek, 2019). Yet, individuals’ creative potential may be also used toward less prosocial, menacing goals. In this regard, the term malevolent creativity has been coined for creative ideas that are generated to purposefully inflict material, mental, or physical harm on others (e.g., Cropley et al., 2008, 2014). Importantly, the explicit motivations to do harm are what distinguishes malevolent creativity from other concepts like negative creativity, where harmfulness just happens to be an unintended by-product of creative ideas (James et al., 1999). To elucidate this difference, *finding creative ways for avoiding office work, likely at the expense of other coworkers*, constitutes an instance of negative creativity (James et al., 1999). By comparison, *deliberately sabotaging the work of a coworker in a creative way in order to keep him or her from getting a promotion* denotes an instance of malevolent creativity. While empirical investigations into this darker side of creativity are still scarce, malevolent creativity on a larger scale is recognized in creative warfare, resourceful crime, and unprecedented acts of terrorism (e.g., Cropley et al., 2008). Yet, malevolent creativity also regularly occurs in decidedly ordinary settings, with researchers listing manipulation and deception, harassment, theft, and revenge as manifestations in daily life (Harris et al., 2013; Harris and Reiter-Palmon, 2015). For the assessment of malevolent creativity as such (i.e., outside the domain of humor), we use a newly developed behavioral test, where participants are encouraged to come up with creative ways to take revenge on/sabotage a wrongdoer [Malevolent Creativity Task (MCT); Perchtold-Stefan et al., under review]. This test specifically captures the fluency of coming up with ideas that are at the same time creative (original) and malevolent. While it deliberately positions creative ideation in a social context and targets a specific type of creativity, the format of the test closely resembles that of prototypical creativity tests.

We propose that, similar to the motivation stage at the beginning of the process of humor creation, in spontaneous general (i.e., non-humorous) creative ideation, the production of creative ideas is initiated by a motivational stage as well. We further propose that similar to humor production, the motivation for creative ideation is contingent on the latent social goals of the individual. Hence, as these latent social goals show in the comic styles, we expected individuals with greater typical use of dark comic styles to be more fluent in producing malevolent creative ideas in a relevant behavioral test. To differentiate effects on malevolent creativity from potential effects on general creative ideation, we also tested if there was a relation between humor motivation and performance on a standard verbal creativity test. Based on previous findings in the domain of humor creation (Heintz, 2019), we expected correlations with general humor motivation (i.e., the inclination to engage in any humor; overlapping variance of several comic styles) or wit. In line with previous investigations with the comic styles, we considered gender as a potential covariate in all analyses, given that men were previously reported to score higher on particularly the darker comic styles of cynicism and sarcasm, but also satire (Ruch et al., 2018). Moreover, previous literature also suggested higher malevolent creativity in men than women (e.g., Lee and Dow, 2011; Harris and Reiter-Palmon, 2015; Perchtold-Stefan et al., under review).

## Materials and Methods

### Participants

In total, 110 participants were recruited for the study. Three participants failed to show up at the agreed appointment, and one participant was excluded from the main analyses after data collection due to non-compliance with test instructions. The final sample comprised *n* = 106 participants (48 men), aged between 18 and 39 years (*M* = 22.67, *SD* = 4.53). Participants were recruited *via* social media and offline *via* posters at several university campuses. The majority of participants were university students enrolled in various fields (*n* = 93), the rest were high school graduates (*n* = 13). The study was approved by the authorized ethics committee. Participants gave their written consent to participate in the study. After receiving general instructions, participants completed the standard creativity test, followed by the malevolent creativity test, and finally, the CSM questionnaire.

### General Creative Potential

The verbal imagination subscales of the well-established German Intelligence Structure Test [Berlin Intelligence Structure Test (BIS); Jäger et al., 1997] were used to assess participants’ general creative potential. Participants completed four different subtests that required them to produce and write down as many different ideas as possible (e.g., alternative uses; constructing as many different sentences with given words as possible) in a limited amount of time (2–2.5 min). Verbal creativity was scored by two trained raters. A total verbal creativity score was computed by adding the number of generated, non-redundant ideas in each subtest (see Jäger et al., 1997). Interrater reliability was ICC = 0.99.

### Malevolent Creativity

**FIGURE 1:**
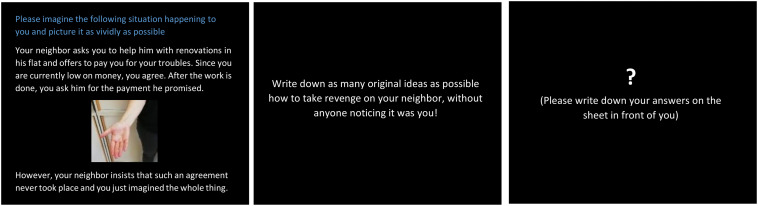
Individuals were presented with a negative social situation for 30 s and subsequently received specific instructions to take revenge/sabotage the wrongdoer (10 s). Then, they were given 3 min to generate and write down as many original ideas as possible as how to deal with the situation. The next situation appeared at the sound of a short tone *via* headphones.

The MCT (Perchtold-Stefan et al., under review) consisted of four realistic, open-ended problems that depicted different sorts of unfair behavior from peers/associates, as it was shown that malevolent creativity is likely to occur in unfair and provocative contexts (e.g., Harris and Reiter-Palmon, 2015; Baas et al., 2019). In the money item of the MCT, participants face the following scenario: *“Your neighbor asks you to help him with renovations in his flat and offers to pay you for your troubles. Since you are currently low on money, you agree. After the work is done, you ask him for the payment he promised. However, your neighbor insists that such an agreement never took place and you just imagined the whole thing.”* Participants were instructed to generate as many original ideas as possible to react to the unfair behavior depicted in these situations in order to get back at or sabotage the wrongdoer. These instructions conform to the definition of malevolent creativity as the generation of novel ideas with the goal to deliberately harm and damage others (e.g., Cropley et al., 2008). A practice item was given prior to the task. Each situation was presented on a computer screen for 30 s and was supplemented by a matching photograph. Participants were told to imagine the situation happening to them and to try and picture it as vividly as possible. Then, a situation-specific instruction for idea generation followed (10 s). Subsequently, at the appearance of a white question mark on the screen, participants then wrote down as many original ways as possible to sabotage that person/take revenge for the unfair treatment on a sheet in front of them. After the allotted time of 3 min, a short tone indicated a new vignette appearing on the screen (for a schematic representation of the MCT, see Figure 1). On average, participants generated a total of *M* = 17.85 ideas (*SD* = 6.95). However, only ideas that met the instructions of being (at least slightly) malevolent were scored as valid (*M* = 91.2% of generated ideas). Malevolence was scored on a 4-point Likert scale by four independent raters, with 1 indicating slight malevolence (e.g., *talking badly with friends about the wrongdoer*) and 4 indicating high malevolence (e.g., *hiring some people to kidnap the wrongdoer and beat some sense into them;* ICC = 0.88). In line with previous studies (Harris et al., 2013; Harris and Reiter-Palmon, 2015), ideas were deemed malevolently creative if they qualified as both malevolent and original. To this end, the same four raters were trained to rate the generated ideas for originality on a 4-point Likert scale (1 = not original, 4 = very original; cf. Consensual Assessment Technique; Amabile, 1982). Interrater reliability was ICC = 0.91. A final score of malevolent creativity was computed using only malevolent ideas with an average originality rating of ≥2 (=moderately original). Thus, in this study, malevolent creativity was operationalized in terms of the number of generated ideas that were both malevolent and original, which served as the main variable of interest.

### Comic Styles

In the CSM questionnaire (Ruch et al., 2018; formerly named 8SHCS), participants are asked to rate the extent to which 48 statements apply to the way they typically express humor on a 7-point Likert scale (from 1 “strongly disagree” to 7 “strongly agree”). It was developed to specifically assess qualitative individual differences in humor production. The questionnaire comprises eight subscales, with six marker items for each comic style. Individuals rate the extent to which they use humor in the form of sarcasm (critical, biting remarks, and schadenfreude; e.g., “Biting mockery suits me”), cynicism (comments that question morality and hypocrisy; e.g., “I tend to show no reverence for certain moral concepts and ideals, but only scorn and derision”), irony (saying the opposite of what is meant, which is only understood by insiders; e.g., “My irony unveils who is smart enough and understands something and who does not”), satire (criticizing inadequacies with the aim to improve them; e.g., “I like to ridicule moral badness to induce or increase a critical attitude in other people”), wit (clever and spontaneous wordplays; e.g., “I surprise others with funny remarks and accurate judgments of current issues, which occur to me spontaneously”), benevolent humor (tolerant, gentle, and forgiving view on weaknesses and mistakes; e.g., “When my humor is aimed at human weaknesses, I include both myself and others”), fun (good-natured jesting; e.g., “I like to make jests and to be silly”), and nonsense (going beyond logical boundaries; e.g., “Humor doesn’t have to make sense; the opposite holds true for me: the more absurd, the funnier”).

Structural analysis of the CSM (Ruch et al., 2018) yielded a second-order factor comprising “dark” comic styles, implicating mock and ridicule, and one or two factors comprising more benevolent or neutral comic styles. Sarcasm and cynicism clearly showed the highest loadings on the “dark” factor. Irony and satire also loaded on this factor, but especially satire had a double loading with the “good humor” factor. Here, it is argued that while satire is aimed at hurting others, there are also positive intentions in this type of corrective humor (Ruch and Heintz, 2016; see also Ruch et al., 2018). The clustering of sarcasm, cynicism, and irony matches the common mean-spirited interpersonal goals attributed to these styles implying intentions of hurting or upsetting (or “laughing at”) others (see also Schmidt-Hidding, 1963; Ruch, 2012). Validity of the overlapping variance of the typical use of sarcasm, cynicism, and irony in terms of latent mean-spirited social goals was confirmed by a targeted neurophysiological study (Papousek et al., 2017). This study also indicated that satire acts differently, thus corroborating the structural analysis in that matter. The commonalities of the lighter comic styles are less well defined (Ruch et al., 2018). Somewhat divergent from the structural analysis, the neurophysiological study that specifically targeted the interpersonal aspects of the comic styles underscored that the lighter styles were more heterogeneous and that only the benevolent humor style was affiliated with unequivocally good-natured (or “laughing-with”) interpersonal goals (Papousek et al., 2017; Ruch et al., 2018). However, this unique position of the benevolent humor scale among the lighter styles is in line with the theoretical conceptualization of the styles in terms of their interpersonal functions. These include also malicious intentions (wit) or less explicit or weaker interpersonal intentions (fun, nonsense) in the remaining lighter styles (see Schmidt-Hidding, 1963; Ruch, 2012).

### Statistical Analysis

On the basis of the extensive psychometric analyses of the CSM, which indicated (also) unique variance of the scales, it was recommended that the major level of analyses should be the level of individual comic styles (Ruch et al., 2018). Therefore, the first step in our statistical analysis was to calculate Pearson correlations between each of the eight **CSM** scales and the psychometric creativity indicators: (1) general creative potential (BIS fluency) and (2) malevolent creativity. Although subsequent analyses focused on the MCT malevolent creativity score (fluency in malevolent creativity), Pearson correlations with the originality and malevolence scores on the MCT are also reported. Our research question for general creative potential (please see introductory section) involves the assessment of overlapping variance among comic styles as well as the potentially unique variance of individual styles, particularly wit. Accordingly, a standard multiple regression analysis was conducted. All eight comic styles in the CSM were simultaneously entered as predictors, with general creative potential (BIS) serving as the dependent variable. Thus, from a significant multiple regression coefficient (*R*), it can be concluded that individuals’ overall use of humor, irrespective of style, explains a significant amount of variance in general creative potential. Further, by looking at zero-order correlations (*r*) and semi-partial correlations (*sr*) obtained in the multiple regression analysis, it can be examined whether potential links to standard creativity were mainly driven by a specific type of humor (*r*), and whether creativity was related to unique variance of using one particular type of humor independently from other types (*sr*).

For our research question concerning individual differences in malevolent creative ideation (2), we took a different approach. Here, the common variance among certain comic styles, which supposedly is attributed to the malicious latent social goals inherent in these styles, was in the focus of interest. Accordingly, a different statistical strategy was required to examine the specific contribution of comic styles affiliated with malicious interpersonal goals. In line with the psychometric–structural and validating findings of previous studies (outlined above; please see section “Comic Styles”), a composite score of the use of humor implicating latent malicious social goals was calculated, comprising the CSM scales sarcasm, cynicism, and irony^1^. In a standard multiple regression analysis, the variance of this score was contrasted with the use of humor based on unequivocally good-natured social intentions (scores on the benevolent humor scale). The statistical assumptions for the models (i.e., ratio of cases to independent variables, normality, independence of errors, homoscedasticity, linearity, and absence of multicollinearity) were met. A significance level of *p* < 0.05 (two-tailed) was used. Supplementary analyses included separate regression analyses for originality and malevolence scores on the MCT (details are reported in the Supplementary Material^2^), tests for potential gender differences (*t*-tests) and potential moderator effects of gender in the multiple regression models. Correlations between general creative potential and malevolent creativity and intercorrelations among CSM scales are reported as well.

## Results

Descriptive statistics for all creativity measures are reported in Table 1, descriptive statistics for the CSM are reported in Table 2.

**TABLE 1:** Descriptive statistics for the behavioral creativity measures.

**Creativity measure**	**α**	***M***	***SD***	**Min**	**Max**
**General creative potential (BIS)**					
Fluency*	0.78	30.92	9.03	15	65
**Malevolent creativity (MCT)**					
Fluency	0.89	16.48	7.52	1	36
Originality	0.71	1.81	0.38	1	3.13
Malevolence	0.66	2.15	0.35	1	2.96
Malevolent creativity*	0.78	5.34	4.38	0	17

α, Cronbach’s alpha; M, mean value; SD, standard deviation; Min, minimum; Max, maximum; N = 106. *Inference statistical analyses focus on the Berlin Intelligence Structure Test (BIS) fluency and the Malevolent Creativity Task (MCT) malevolent creativity score. Detailed analyses for the other scores are reported in the Supplementary Material.

**TABLE 2:** Descriptive statistics for the CSM.

**Comic styles**	**α**	***M***	***SD***	**Min**	**Max**
Benevolent humor	0.69	5.10	0.93	1.83	6.83
Fun	0.80	4.45	1.09	1.17	6.67
Wit	0.78	4.68	0.97	2	6.5
Nonsense	0.87	4.88	1.27	1.5	7
Irony	0.75	4.44	1.08	1.33	6.67
Sarcasm	0.79	3.40	1.27	1	6
Satire	0.78	4.12	1.11	1.5	7
Cynicism	0.85	3.80	1.40	1	7
Dark humor (irony, sarcasm, cynicism)	0.70	3.88	1.00	1.5	6.56

**α, Cronbach’s alpha; M, mean value; SD, standard deviation; Min, minimum; Max, maximum; CSM, Comic Style Markers; N = 106.**

### General Creative Potential

General humor motivation, i.e., the inclination to produce any humor, irrespective of specific style and affiliated social goals, accounted for 19% of variance in general creative potential [*R*^2^ = 0.19; *F*(8, 97) = 2.83, *p* = 0.007]. Individual zero-order and semi-partial correlations revealed that this relationship pertained to the comic style of wit in particular (*sr* = 0.40, *p* < 0.001). Zero-order correlations and the results of the multiple regression analysis are reported in Table 3.

**TABLE 3:** Correlations between the typical display of different types of humor (CSM) and the general creative potential measured in a behavioral test (BIS).

	***R*^2^**	***r***	***p*(*r*)**	**sr**	***p*(sr)**
Benevolent humor	0.19	<0.01	0.930	−0.14	0.122
Fun		0.14	0.164	0.07	0.466
Wit		**0.33**	**<0.001**	**0.40**	**<0.001**
Nonsense		0.08	0.443	0.08	0.407
Satire		0.06	0.539	−0.10	0.274
Irony		0.05	0.594	−0.14	0.127
Sarcasm		0.04	0.675	0.05	0.600
Cynicism		0.07	0.502	0.12	0.213

**Standard multiple regression analysis; F(8, 97) = 2.83, p = 0.007. R^2^, proportions of variance explained by the model in total; r, Pearson correlation; sr, semi-partial correlation; CSM, Comic Style Markers. Significant correlations are highlighted in bold font.**

### Malevolent Creativity

Table 4 shows the zero-order correlations between typical use of individual comic styles and fluency in producing malevolent creative ideas. Correlations were significant for all comic styles presumably affiliated with malicious social goals (cynicism, *r* = 0.33, *p* < 0.001; sarcasm, *r* = 0.20, *p* = 0.042; irony, *r* = 0.20, *p* = 0.043), including satire, which is more ambivalent in terms of social goals (*r* = 0.24, *p* = 0.015). Additionally, correlations with originality and malevolence scores are reported, mirroring the observed pattern with the fluency-based malevolent creativity index. The multiple regression analysis contrasting the aggregated use of malicious comic styles with the use of benevolent humor confirmed the specific contribution of comic styles affiliated with malicious interpersonal goals (*sr* = 0.28, *p* = 0.004; benevolent humor: *sr* = 0.001, *p* = 0.979; Table 5). Supplementary regression analyses for originality and malevolence scores on the MCT yielded a similar pattern of results (originality: *sr* = 0.23, *p* = 0.019; malevolence: *sr* = 0.29, *p* = 0.003; see Supplementary Material for details). The correlation between the habitual use of humor with malicious goals and humor with benevolent goals was *r* = 0.36 (*p* < 0.001).

**TABLE 4:** Correlations between the typical display of different types of humor (CSM) and the malevolent creativity measured in a behavioral test (MCT).

	**Malevolent creativity**		**Originality**		**Malevolence**	
	***r***	***p*(*r*)**	***r***	***p*(*r*)**	***r***	***p*(*r*)**
Benevolent humor	0.11	0.250	−0.02	0.825	0.02	0.861
Fun	0.18	0.062	0.12	0.242	0.09	0.383
Wit	0.16	0.113	0.08	0.404	−0.12	0.223
Nonsense	0.18	0.073	0.14	0.152	0.07	0.470
Satire	**0.24**	**0.015**	0.16	0.103	0.18	0.071
Irony	**0.20**	**0.043**	0.15	0.136	0.15	0.122
Sarcasm	**0.20**	**0.042**	0.10	0.308	**0.21**	**0.034**
Cynicism	**0.33**	**<0.001**	**0.23**	**0.017**	**0.28**	**0.004**

**Significant zero-order correlations are highlighted in bold font (p < 0.05). CSM, Comic Style Markers; MCT, Malevolent Creativity Task.**

**TABLE 5:** Correlations between the typical display of humor affiliated with latent malicious social goals and the humor affiliated with benevolent goals (CSM) with malevolent creativity (MCT).

	***R*^2^**	***r***	***p*(*r*)**	**sr**	***p*(sr)**
Humor with malicious goals	0.10	**0.31**	**0.001**	**0.29**	**0.002**
Humor with benevolent goals		0.11	0.250	<0.01	0.964

**Standard multiple regression analysis; F(2, 103) = 5.58, p = 0.005. R^2^, proportions of variance explained by the model in total; r, Pearson correlation; sr, semi-partial correlation; CSM, Comic Style Markers; MCT, Malevolent Creativity Task. Significant correlations are highlighted in bold font.**

### Supplementary Analyses

General creative potential and malevolent creativity were positively correlated at *r* = 0.37 (*p* < 0.001). Men showed higher malevolent creativity on the MCT than women [men: *M* = 6.44, *SD* = 4.13; women: *M* = 4.62, *SD* = 4.49; *t*(104) = 2.15, *p* = 0.034]. There were no significant gender differences in general creative potential [*t*(104) = -0.69, *p* = 0.491]. Men also reported greater use of satire [men: *M* = 3.46, *SD* = 1.07; women: *M* = 2.84, *SD* = 1.08; *t*(104) = 2.96, *p* = 0.004] and cynicism than women [men: *M* = 3.21, *SD* = 1.53; women: *M* = 2.48; *SD* = 1.21; *t*(104) = 2.74, *p* = 0.007], with no gender differences emerging for the other comic styles (all *p*s > 0.074). Adding gender to the regression analyses did not change the previous pattern of results and yielded no significant interaction terms (all *p*s > 0.251). Intercorrelations among the CSM scales are depicted in Table 6.

**TABLE 6:** Intercorrelations among CSM scales.

	**Ben. humor**	**Fun**	**Wit**	**Nonsense**	**Satire**	**Irony**	**Sarcasm**	**Cynicism**
Ben. humor	–							
Fun	0.34**	–						
Wit	0.43**	0.30**	–					
Nonsense	0.52**	0.36**	0.20*	–				
Satire	0.48**	0.30**	0.51**	0.38**	–			
Irony	0.34**	0.19	0.34**	0.30**	0.44**	–		
Sarcasm	0.23*	0.22*	0.37**	0.19	0.51**	0.45**	–	
Cynicism	0.33**	0.20*	0.19	0.43**	0.58**	0.38**	0.50**	–

**p < 0.01, *p < 0.05. CSM, Comic Style Markers.

## Discussion

The present study investigated the assumption that humor and general (non-humorous) creativity do share not only cognitive but also motivational aspects. We proposed that the motivation to initiate (socially relevant) creative ideation may be contingent on the latent social goals of the individual, given that such goals also influence the initiation of humor creation (Feingold and Mazzella, 1993; Papousek et al., 2017). In line with this idea, the present study found that individuals with greater typical use of comic styles that are affiliated with malicious interpersonal goals such as hurting or upsetting others or demonstrating superiority (Ruch, 2012; Papousek et al., 2017) were more fluent in producing malevolent creative ideas in a relevant, not humor-related psychometric test. Thus, individual differences in humor motivation do not only translate to the productivity in creating relevant types of humor as was shown previously (Perchtold et al., 2019). They also seem to relate to the capacity of coming up with creative ideas outside the domain of humor. Importantly, while our composite score for malevolent creativity was fluency-based, we are confident in interpreting these results in terms of a broader capacity for malevolent creativity, since correlations of humor reflecting malicious interpersonal goals also extended to quality indicators of malevolent creativity, i.e., originality and malevolence of generated ideas.

Additional evidence corroborating this shared motivation to inflict harm may be derived from studies that find antagonistic personality traits and behavior aimed at manipulating and dominating others quite robustly linked to both malicious humor (Martin et al., 2012; Proyer et al., 2012; Zeigler-Hill et al., 2016; Vrabel et al., 2017) and malevolent creativity (Lee and Dow, 2011; Harris and Reiter-Palmon, 2015; Perchtold-Stefan et al., under review). Beyond that, it was reported that individuals scoring higher on dark comic styles in the CSM found it more acceptable to laugh at “taboo” topics and social groups that are vulnerable to prejudice, reflecting their propensity to purposefully break with recognized norms and values (Mendiburo-Seguel and Heintz, 2019). This motivation for overriding social standards is a likely catalyst for increased malevolent creativity in response to provocative interpersonal situations like those depicted in the MCT.

In this regard, it seems important to note that while the MCT is a laboratory-bound, performance-based instrument, Perchtold-Stefan et al. (under review) underlined its ecological validity in terms of everyday behavior. The authors showed that individuals with a higher MCT performance also reported more malevolent creativity behaviors in daily life, like playing tricks on others, lying, or hurting others in creative ways (assessed with the Malevolent Creativity Behavior Scale; Hao et al., 2016). This lends support to the idea that latent intentions to achieve certain interpersonal goals in part also drive creative ideation and behavior in daily life.

Greater fluency in producing malevolent creative ideas was not only observed in participants scoring higher on the comic styles most clearly affiliated with malicious social goals (cynicism, sarcasm, irony; Ruch, 2012; Papousek et al., 2017; Ruch et al., 2018) but also in those with higher scores on satire. The use of satire-type humor is more ambivalent in terms of social goals because while satire is aimed at correcting the transgressions of others, positive intentions may be included in the use of corrective humor as well (Ruch and Heintz, 2016; Ruch et al., 2018). As the intention to correct the transgressions of others was prevalent in the revenge-oriented instructions of the MCT, it may have augmented the correlation with the self-reported typical use of satire. Greater or lesser typical display of other comic styles was clearly not related to malicious creativity. This also holds for benevolent humor, the social goals of which may be seen as opposite to those of the darker comic styles. This detail underlines the conceptualization of the comic styles in terms of humor motivation and humor use rather than of just expressions of one bipolar dimension of preference and provides further support for the independent value of the styles (Ruch et al., 2018).

Our results clearly showed that the above discussed relationships do not pertain to general creative potential. The pattern of correlations between humor motivation and performance on the standard creativity test was very much different from that of malicious creativity. Yet, individuals with a greater inclination to create humor in daily life also produced a higher number of creative ideas in the standard verbal creativity test. At first view, this seems to align with previous studies that suggested higher creative potential in individuals with greater endorsement of humor in daily life (Chang et al., 2015; also see Kellner and Benedek, 2017). However, the statistical results clearly show that the relationship between typical use of humor and general creative potential was driven by one particular comic style only: wit. In previous research, higher scores on wit were linked to greater productivity in creating humor in a cartoon caption task (Heintz, 2019). There is some indication that this and the present result are attributed to the same more cognitively and ability-based links. Wit is characterized by clever wordplays, typically with a surprising punch line that uses unusual combinations created on the spot, and the creation of wit is considered most cognitively challenging of all comic styles (Ruch et al., 2018), up to the point that the use of wit was often conceptualized close to an ability in earlier research (Heintz, 2019). Creation of wit also seems most akin to the out-of-the-box thinking that is inherent to creative ideation, perhaps down to the interplay of spontaneous and controlled processes in creative cognition. It may be presumed that in wit, diffusively and weakly connected concepts are linked in a rapid and automatic manner and are then skillfully integrated in order to create a surprising, comical effect (for an overview of these processes in creativity, see Benedek and Jauk, 2018). Moreover, wit was the only comic style positively correlated with verbal intelligence (Ruch et al., 2018). Creative ideation has been found to involve various verbal ability demands such as associative abilities assessed in word association tasks (e.g., Benedek et al., 2012) or broad retrieval ability measured by means of various verbal fluency tests (e.g., Silvia et al., 2013; Forthmann et al., 2019). Likewise, verbal intelligence-related demands are also implicated in the capability to create humor (as assessed by a cartoon caption task; Greengross and Miller, 2011). Accordingly, it seems likely that verbal intelligence-related demands could operate as a mediating factor in the relationship between the use of wit, humor production ability, and creativity. Thus, these results further add to the notion of shared cognitive processes in the production of humor and creative ideation.

A limitation of the present research is that the multiple regression analysis for different comic styles and general creative potential had a rather low n:k ratio (about 13:1), which raises potential concerns about statistical power and stability of the regression coefficients. Future studies are warranted to test these relationships in larger samples. Additionally, our findings may only generalize to young, well-educated adults, necessitating replication with more representative samples of different ages and educational backgrounds. This study emphasized the motivational components and social goals embedded in the use of certain comic styles. However, comic styles also reflect interindividual differences in humor-related affect, behaviors, and cognition (e.g., Ruch et al., 2018; Heintz, 2019). Thus, it cannot be excluded that the results of the present study partly reflect joint effects of these humor components. Next, the present study focused on one particular class of latent social goals only. Future research may evaluate similar relationships for the production of creative ideas in different social contexts, for instance, ones that prompt pro-social ideas. As a further limitation, ideational fluency was used as the only indicator for general creative potential, since the employed test focused on fluency and diversity instead of originality of ideas (Jäger et al., 1997). While fluency qualifies as a valid indicator of creativity (e.g., Jauk et al., 2014), future related studies should use creativity measures that also emphasize originality of ideas (e.g., alternate uses task), given that creative potential is best estimated by multiple indices (Runco and Acar, 2012). Lastly, given the correlational design of this study, causality and direction of influences cannot be directly inferred and need to be tested in longitudinal investigations.

Taken together, in considering latent social goals embedded in the typical use of humor, the present study offers a novel perspective as to how motivational factors may link the use of specific types of humor to specific types of creativity. Given the social interpretation of the motivational aspects, the findings also provide empirical support to the view that similar social processes are involved in humor creation and non-humorous creative ideation (O’Quin and Derks, 1997). Overall, though preliminary, our findings suggest that creativity research may benefit from the inclusion of motivational mechanisms that have long been recognized in conceptualizations of humor.

## Data Availability Statement

The raw data supporting the conclusions of this article will be made available by the authors, without undue reservation, to any qualified researcher.

## Ethics Statement

The studies involving human participants were reviewed and approved by the Ethics Committee of the University of Graz. The patients/participants provided their written informed consent to participate in this study.

## Author Contributions

CP-S, IP, and AF conceptualized the study. CP-S collected, analyzed, and interpreted the data. CP-S and IP drafted the manuscript. AF and CR critically reviewed the manuscript. All authors gave their final approval of the manuscript.

## Conflict of Interest

The authors declare that the research was conducted in the absence of any commercial or financial relationships that could be construed as a potential conflict of interest.

## Footnotes

1 The selection of scales for this composite score was guided by previous research. We purposely refrained from pursuing a data-driven approach.

2 The Supplementary Material also includes intercorrelations between all indices of malevolent creativity, as well as correlations between comic styles and fluency of generated ideas in the MCT.
