# Supplementary material for: Motivational Factors in the Typical Display of Humor and Creative Potential: The Case of Malevolent Creativity
Source: Front Psychol. 2020 Jun 23;11:1213. doi: 10.3389/fpsyg.2020.01213 (PMC7325593; doi:10.3389/fpsyg.2020.01213)
Supplement: Supplementary file 1 [file Table_1.DOCX]

**Supplementary material**

*Table A: Correlations between different comic styles (CSM) and fluency of generated ideas in the malevolent creativity test (MCT)*

|  | Fluency (MCT) | |
| --- | --- | --- |
|  | r | p (r) |
| Benevolent Humor | .15 | .117 |
| Fun | .19 | .052 |
| Wit | **.22** | **.025** |
| Nonsense | .04 | .705 |
| Satire | **.25** | **.010** |
| Irony | **.30** | **.002** |
| Sarcasm | **.20** | **.039** |
| Cynicism | **.25** | **.010** |

Note: Significant zero-order correlations are highlighted in bold font (p <.05).

*Table B: Intercorrelations between all creativity measures*

|  | Malevolent Creativity (MCT) | Fluency (MCT) | Originality (MCT) | Malevolence (MCT) | Fluency (BIS) |
| --- | --- | --- | --- | --- | --- |
| Malevolent Creativity (MCT) | - |  |  |  |  |
| Fluency (MCT) | .61** | - |  |  |  |
| Originality (MCT) | .67** | .28** | - |  |  |
| Malevolence (MCT) | .55** | .38** | .50* | - |  |
| Fluency (BIS) | .37** | .27** | .15 | -.10 | - |

Note: ** p <.01, * p <.05.

*Table C: Correlations between typical use of humor affiliated with latent malicious social goals and humor affiliated with benevolent goals (CSM) with originality in malevolent creativity (MCT)*

|  | R² | r | p (r) | sr | p (sr) |
| --- | --- | --- | --- | --- | --- |
| Humor with malicious goals | .05 | **.20** | **.036** | **.23** | **.019** |
| Humor with benevolent goals |  | -.02 | .825 | -.11 | .273 |

Note: Standard multiple regression analysis; *F(*2,103) = 2.88, *p* = .061;
R² = proportions of variance explained by the model in total, r = Pearson correlation; sr = semipartial correlation. Significant correlations are highlighted in bold font.

*Table D: Correlations between typical use of humor affiliated with latent malicious social goals and humor affiliated with benevolent goals (CSM) with malevolence in malevolent creativity (MCT)*

|  | R² | r | p (r) | sr | p (sr) |
| --- | --- | --- | --- | --- | --- |
| Humor with malicious goals | .08 | **.27** | **.005** | **.29** | **.003** |
| Humor with benevolent goals |  | .02 | .861 | -.09 | .336 |

Note: Standard multiple regression analysis; *F(*2,103) = 4.65, *p* = .012;
R² = proportions of variance explained by the model in total, r = Pearson correlation; sr = semipartial correlation. Significant correlations are highlighted in bold font.
